# ILPC: simple chemometric tool supporting the design of ionic liquids

**DOI:** 10.1186/s13321-016-0152-4

**Published:** 2016-08-19

**Authors:** Maciej Barycki, Anita Sosnowska, Magdalena Piotrowska, Piotr Urbaszek, Anna Rybinska, Monika Grzonkowska, Tomasz Puzyn

**Affiliations:** Laboratory of Environmental Chemometrics, Faculty of Chemistry, Institute for Environmental and Human Health Protection, University of Gdańsk, Wita Stwosza 63, 80-308 Gdańsk, Poland

**Keywords:** Ionic liquids, PCA, Design, Computational, Physicochemical properties, Chemometric

## Abstract

**Background:**

Ionic liquids (ILs) found a variety of applications in today’s chemistry. Since their properties depend on the ions constituting particular ionic liquid, it is possible to synthetize IL with desired specification, dependently on its further function. However, this task is not trivial, since knowledge regarding the influence of particular ion on the property of concern is crucial. Therefore, there is a strong need for new, fast and inexpensive methods supporting the process of ionic liquids’ design, making it possible to predefine IL’s properties even before the synthesis.

**Results:**

We have developed a simple tool (called Ionic Liquid PhysicoChemical predictor: ILPC) that allows for the simultaneous qualitative prediction of four physicochemical properties of ionic liquids: viscosity, n-octanol–water partition coefficient, solubility and enthalpy of fusion. By the means of Principal Component Analysis, we studied 172 ILs and defined distribution trends of those four properties, dependently on the ILs structures. We proved that the qualitative prediction of mentioned properties could be performed on the basis of most simple information we can deliver about ILs, which are their molecular formulas.

**Conclusions:**

Created tool presented in this paper allows fast, pre-synthesis screening of ILs, with the omission of any experimental steps. It can be helpful in the process of designing ILs with preferred properties. We proved that the information encrypted in molecular formula of ionic liquid could be a valuable source of knowledge regarding the IL’s viscosity, n-octanol–water partition coefficient, solubility and enthalpy of fusion. Moreover, we proved that the influence of both ions, constituting the IL, on each of those four properties indicates same, additive trend.

**Graphical Abstract:**
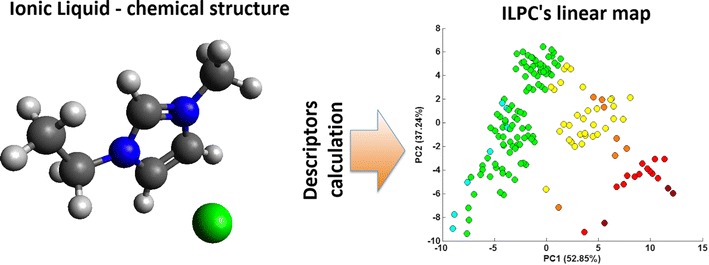
Schematic representation of ILPC performance - the exact position of the ionic liquid on the linear map is determined by its chemical structure

**Electronic supplementary material:**

The online version of this article (doi:10.1186/s13321-016-0152-4) contains supplementary material, which is available to authorized users.

## Background

Ionic Liquids (ILs) are commonly used in a variety of modern applications, with their popularity continuing to increase. In the past several years, we have witnessed a significant step forward in the field of their application [1–9]. Moreover, the amount of available data regarding ILs properties has increased significantly, as has our understanding of their toxicity and behavior in the environment [10–17]. Nevertheless, the field of ILs properties still requires a lot of research, so that the use of those compounds can be considered thoughtful and safe. In our approach, we try to contribute to the increase of this knowledge by the means of chemometrical analyses.

One of the most important aspects of the phenomenon of widespread ILs’ use is that the properties of these materials can be adjusted to the needs of the specific application. Depending on the specific ions that we choose as the constituents of the IL, we can synthesize a variety of ILs with different characteristics and properties. This is one of the most promising aspects of the chemistry of ILs. However, the ability to adjust their properties for the desired purpose is only possible after a series of experiments determining which structural features are responsible for the selected property. These types of screening tests are very time-consuming and expensive. To overcome this problem, we must develop and apply comprehensive computational tools for quick, cheap and rational assessment of the IL’s properties and risk posed to the environment by novel ILs. It is worth noting that with increasing knowledge of the behavior of ILs, a significant opportunity for theoretical screening studies is currently being created. Blank spots, which are still widely present on the map of our knowledge of ILs, can slowly be filled by observations and pattern analysis, coming from the statistical methods of computational chemistry [18].

The chemometric approach to this matter assumes the analysis of existing data and the use of the results for the systematization of knowledge or even for the prediction of nonexistent data. In our work, we mainly focus on the relationship between the structural features and unique properties of ILs. Using chemometric analysis, we can provide a large amount of useful information, and most importantly, we can identify which aspect of the structure is responsible for the IL property or behavior of interest. In some cases, we can even identify how modification of the structure impacts the property of interest.

In our previous work, we successfully applied a similar approach to determine the relationship between ILs’ structure and their toxicological potential [19]. Our earlier findings allowed us to note several structural features that are crucial for the toxicity of ILs against different organisms. In this work, we have focused on four different physicochemical properties: the viscosity (η), n-octanol–water partition coefficient (K_OW_), water solubility (S) and enthalpy of fusion (**Δ**H_F_), and their relationship with the ILs’ structure.

Ability of predefining ILs properties (both physicochemical and toxicological) can considerably reduce both costs and time of synthetizing new, safe and well performing ionic liquids. Knowledge that can be obtained as a result of theoretical predictions can significantly affect the process of designing new, safer ionic liquids, with properties more accurately corresponding to users needs.

In this work we have attempted to create a simple tool for the determination of IL properties based on their structures (ILPC—Ionic Liquids PhysicoChemical predictor). Information delivered by our tool can be further used in the considerations about particular ILs’ application and safety.

## Results and discussion

**Fig. 1:**
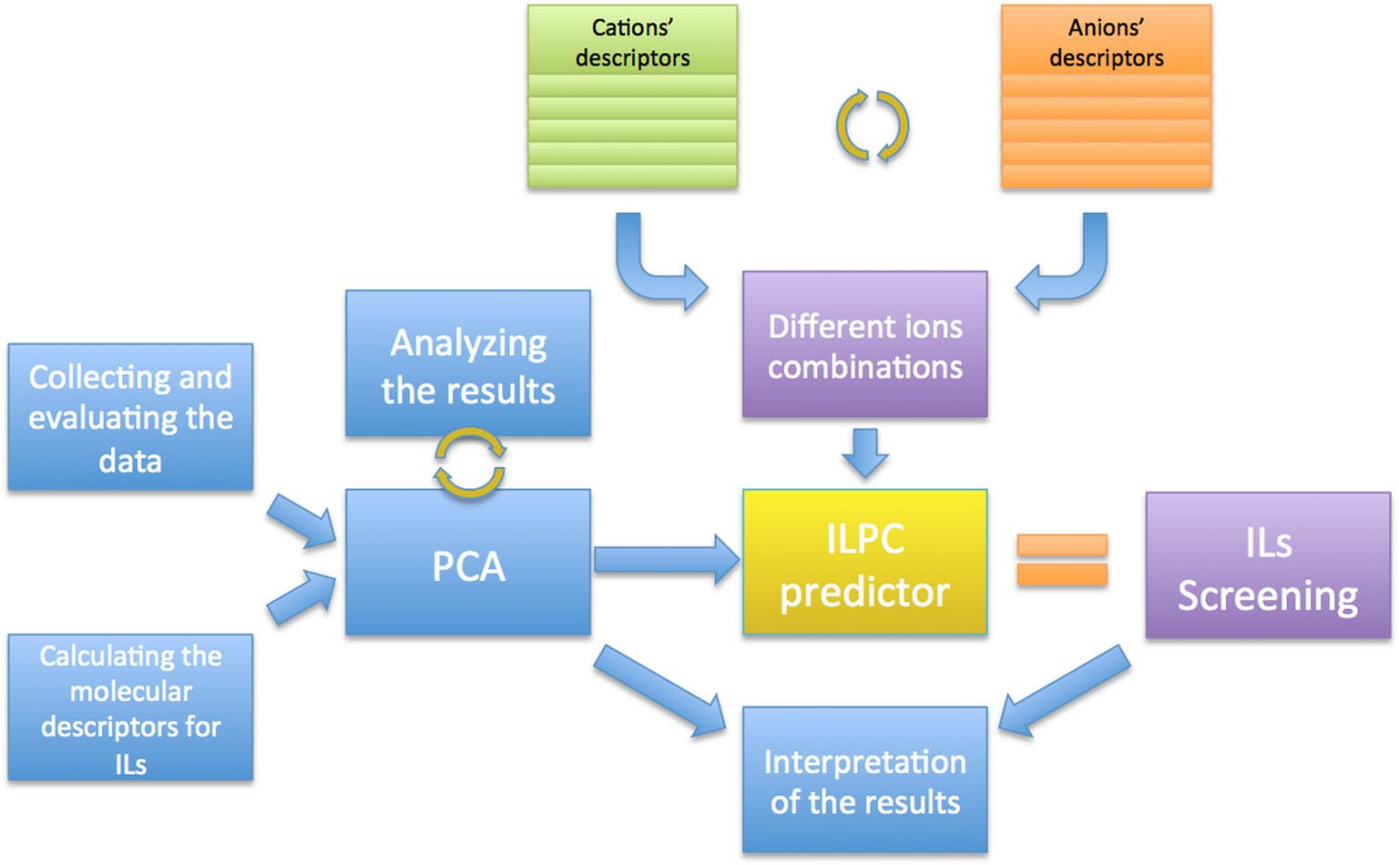
Schematic workflow

In order to develop a tool capable of predicting IL’s physicochemical properties, we conducted a series of pre steps essential for the tool’s development. Initial stages included data collecting as well as translating the structural features of ionic liquids into the simple and mathematically expressible form. Than, we performed a series of PCA analyses, which were in fact the most crucial for the ILPC performance. The workflow is presented on the scheme (Fig. 1).

The main idea of ILPC predictor is bases on the ability of quick and efficient analysis of ILs’ properties, depending on their structural diversity. Finding the relationship between those two factors is therefore crucial for the tool’s development. Employing PCA technique seemed to be a promising way of reducing the informational excess with simultaneous definition of structural features responsible for IL’s physicochemical properties.

The first step of our work was to explore the “chemical space” of structurally diversified ILs (Table 1) and to identify the structural features of anions and cations that are responsible for the observed physical/chemical properties: η, K_OW_, S and **Δ**H_F_. Then, based on the identified relationships, we developed a practical tool for estimating IL properties based on their structure.

**Table 1:** Physicochemical properties investigated in this study; each column contains the number of data points collected for each ILs’ class along with the overall sum of data points

	Viscosity (cPa)	K_OW_ (−)	Solubility (g/L)	Enthalpy of fusion (kJ/mol)
Imidazolium	20	20	6	7
Ammonium	24	7	1	4
Phosphonium	46	0	0	0
Pyridinium	23	1	8	13
Pyrrolidinium	5	0	1	1
Sulfonium	8	0	3	5
Sum	126	28	19	30

We decided to include ILs from an array of common structural sub-groups (e.g., imidazolium, ammonium, phosphonium, pyridinium, pyrrolidinium, sulfonium) for each analysis because we wanted to ensure the global relevance of our results. This means that the model should describe the general behavior of ILs rather than the behavior of specific IL sub-groups. Moreover, having a wider set of diversified compounds increases the probability of identifying significant trends in the properties that might be structure-dependent.

### How are the studied ILs distributed in the space of their structural descriptors?

We started by exploring the distribution of the studied ILs in the multidimensional space of their chemical features, described by molecular descriptors. Molecular descriptors are used to numerically express various aspects of cation and anion structures (e.g., numbers of atoms, bonds, substituents, molecular size, shape, etc.). As such, every IL is described by a series of molecular descriptors. These data can be compiled within a table, in which rows represent particular ILs and columns represent descriptors. The same can be presented as a scatter plot, in which every single point represents one ionic liquid and the values of molecular descriptors are the Cartesian coordinates along the particular dimensions (descriptors). Ionic liquids that possess similar structures are located close to each other and may form small groups or so-called “clusters.”

**Fig. 2:**
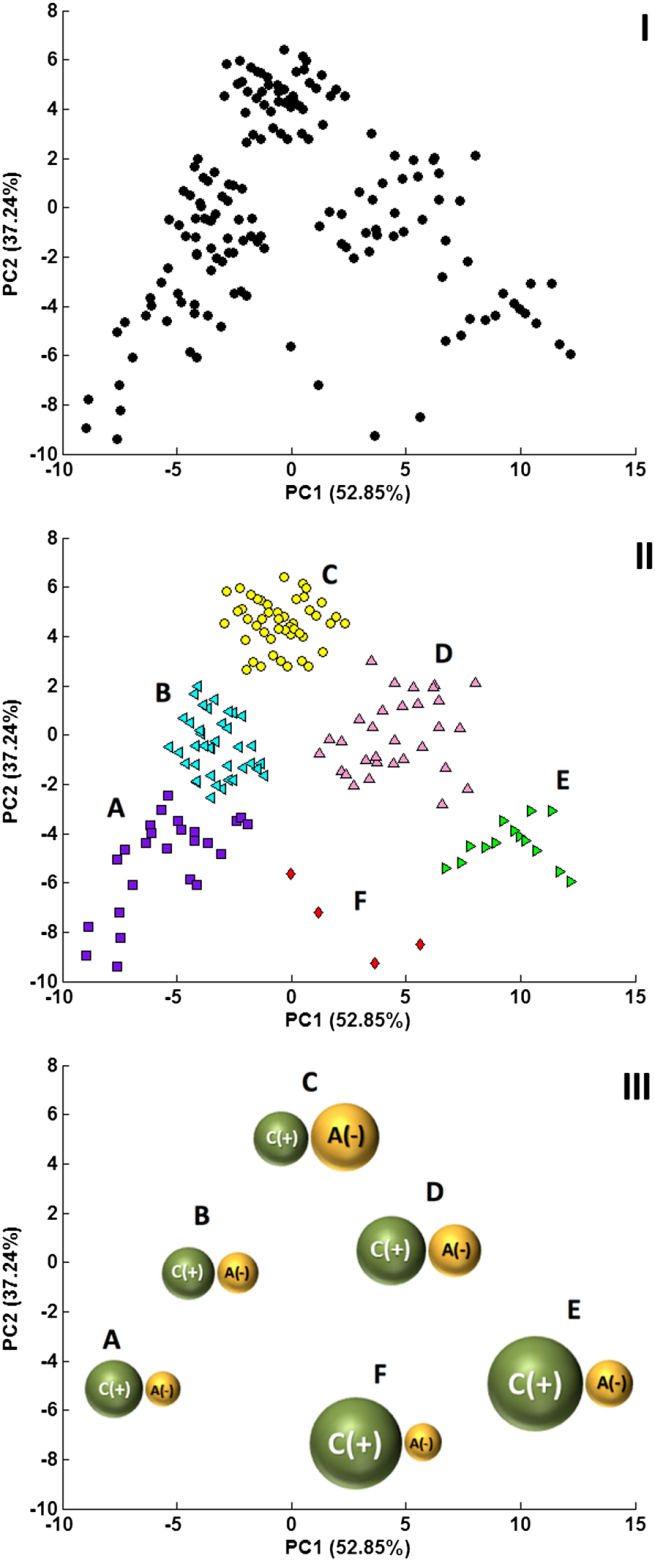
Three scatter plots representing 172 ILs in the space of two PCs. **I** Structural similarities among ILs in the space of constitutional indices. **II** Clusters of ILs determined within the space of constitutional descriptors. **III** Scheme of the structure/size of ILs in particular clusters within the space of two PCs

First, we performed a series of PCA analyses for the entire set of 172 ILs with the entire range of cation descriptors and anion descriptors (1462 for each ion), to find the most diversified distribution of ILs. However, the results were not satisfactory, so we decided to reduce the descriptors set. Therefore, we performed further PCA analyses for each of 19 groups of descriptors from Table 2 (using both cation and anion descriptors). The constitutional indices (82 descriptors) gave the best results for all studied ILs (Fig. 2:I), diversifying them into easily interpretable clusters (see the following paragraph) identified with use of Hierarchical Cluster Analysis (see the Additional file 1 for the detailed results). We expected such diversification to be very useful in the further analysis of ILPC results, as all the clusters had unique and noticeable characters (see the following paragraph).

**Table 2:** Groups and numbers of calculated descriptors for each ion [58]

Group	Number of cation descriptors	Number of anion descriptors	Total
Constitutional indices	41	41	82
Ring descriptors	18	18	36
Topological indices	69	69	138
Walk and path counts	44	44	88
Information indices	46	46	92
2D autocorrelations	149	149	298
Burden eigenvalues	64	64	128
Geometrical descriptors	26	26	52
RDF descriptors	150	150	300
3D MoRSE descriptors	160	160	320
WHIM descriptors	82	82	164
GATEWAY descriptors	208	208	416
Randic molecular profiles	41	41	82
Functional group counts	26	26	52
Atom-centered fragments	36	36	72
Atom-type E-state indices	36	36	72
CATS 2D	62	62	124
2D atom pairs	193	193	386
3D atom pairs	11	11	22
Sum	1462	1462	2924

At this stage, we attempted to determine for which group of descriptors (and by that we mean for which particular structural properties) the distribution of ILs on the PC scatter plot is in good accordance with the distribution of the physicochemical property values (marked as colored dots; see the methodology paragraph for details). In this case, constitutional indices also provided very good results for all studied properties (Fig. 2:I). This group of descriptors turned out to be the most useful for the purpose of ILPC tool development, both in terms of diversifying ionic liquids and underlying the correlation of their structure and properties. Thus, we continued our analysis by taking only this set of results into account.

### How are ILs distributed in the space of constitutional descriptors? The general results of PCA

While developing the ILPC, we focused on the fact that all the findings concerning ILs’ distribution on the linear map should be easily interpretable, in order to provide better understanding of the observed phenomenon of structure property relationship. Therefore, the next step was to interpret the particular PCs while trying to classify the studied ILs based on their structural similarities using constitutional descriptors (82 indices in total). A plot showing the distribution of all 172 ILs in the space of the first two PCs is presented in Fig. 2:I. The first PC (PC1) explains 52.85 % of the variation described by the original set of variables, whereas the second PC (PC2) explains 37.24 %. Thus, as a result of the PC analysis, we reduced the space of the original 82 variables describing ILs to only 2, preserving 90.09 % of the initial variation.

**Fig. 3:**
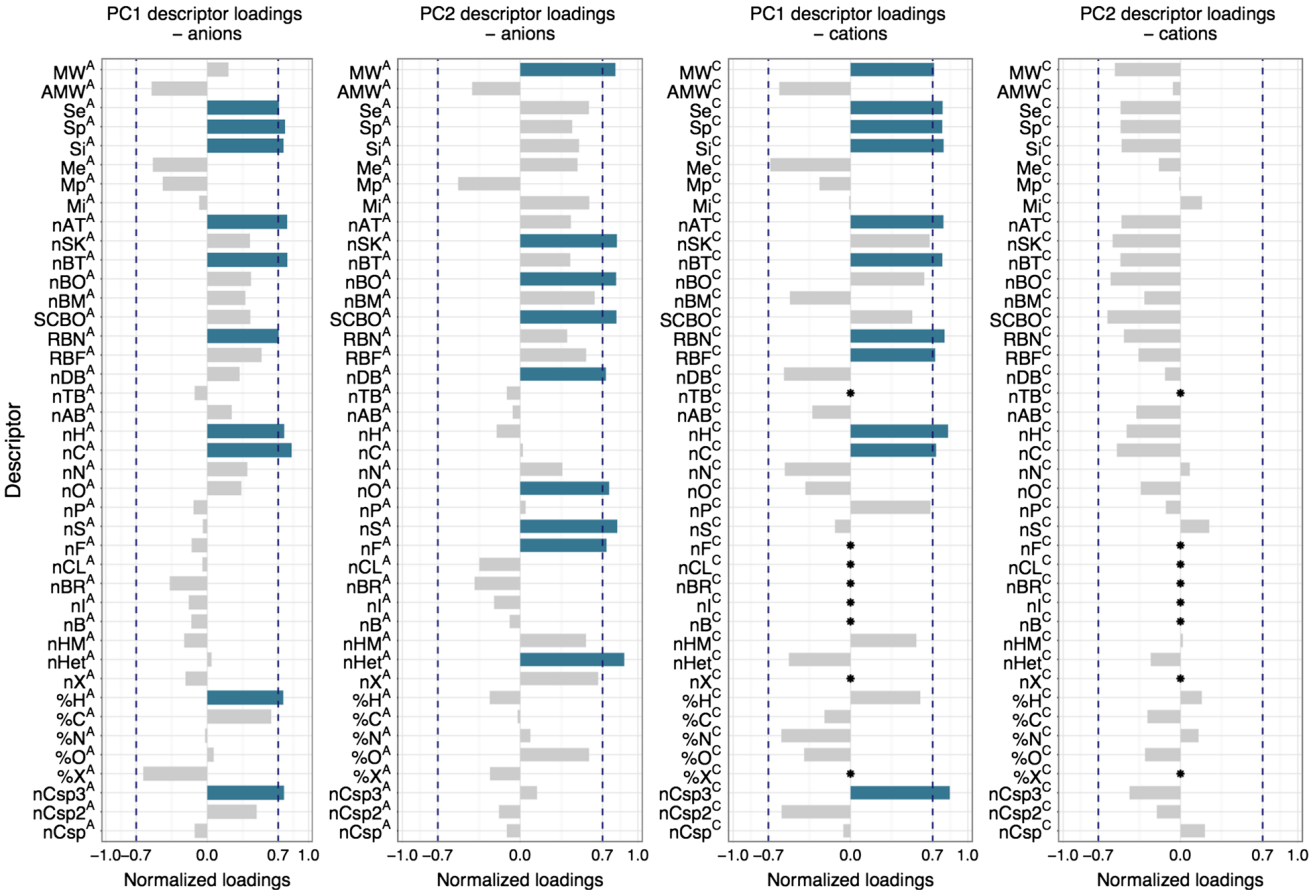
Correlation coefficients between a particular constitutional descriptor and either PC1 or PC2. Values marked in *blue* are significant in particular PCs and determined the physical interpretation of these PC. * The correlation coefficient could not be calculated because the values of the molecular descriptors were constant

As described in the Methodology section, a PC is a linear combination of the original variables, which have different contributions to the PC’s final form. According to Malinowski’s rule, if the absolute value of the correlation between the original variable and the PC (normalized loading) exceeds 0.7, this variable is considered to have a significant influence on the form of the PC. In this manner, one can give a physical interpretation of the PC. The contribution of each variable describing a particular PC in this work is presented in Fig. 3.

Along with the loading values, we determined the descriptors that have the most influence on the first PC. There were nine descriptors referring to cations and anions (Se^C^, Se^A^, Sp^C^, Sp^A^, Si^C^, Si^A^, nAT^C^, nAT^A^, nBT^C^, nBT^A^, RBN^C^, RBN^A^, nH^C^, nH^A^, nC^C^, nC^A^, nCsp^3C^ and nCsp^3A^), two descriptors referring to cations (MW^C^ and RBF^C^) and one referring to anions (H %^A^). All correlation coefficients are positive; therefore, the value of PC1 increases with an increase in the descriptors’ values.

In general, the results indicate that first principal component (PC1) represents the size of the ions in the liquid. It is best seen in case of cation’s descriptors, in which we observe a high correlation between the PC1 and mass defining MW^C^ (molecular weight) descriptor. The descriptors nAT^C^/nAT^A^ (the total number of atoms), nBT^C^/nBT^A^ (the total number of bonds), RBN^C^/RBN^A^ (the total number of rotatable bonds), nH^C^/nH^A^ (the total number of hydrogen atoms) and nC^C^/nC^A^ (the total number of carbon atoms) are also closely related to the size of the particles. Additionally, RBN^C^/RBN^A^ descriptors along with RBF^C^ descriptor (rotatable bonds fraction) also indicate the level of saturation in the particles. The descriptors Se^C^, Se^A^, Sp^C^, Sp^A^, Si^C^ and Si^A^ describe the cumulative electronegativity, polarizability and ionization potential of the atoms that constitute the molecules. They are also directly related to ionic size. In addition, these descriptors differentiate ions with similar size, distinguishing them by their constituent atoms. PC1 values are higher for ions consisting of atoms that have higher polarizability, electronegativity and ionization potential. Since nC^C^/nC^A^ and nH^C^/nH^A^ descriptors also have a large contribution to the form of PC1, we can conclude that PC1 value will be higher for ILs consisting of organic ions. In case of anions, it is also proven by the contribution of H %^A^ (percentage of H atoms) descriptor. The last descriptors with a significant impact on the PC1 form are nCsp^3C^ and nCsp^3A^ (the total number of carbon atoms of sp^3^ hybridization, for both cations and anions). Thus, PC1 will be higher for saturated compounds than for unsaturated or ring-containing compounds.

In contrast, the second principal component (PC2) includes only anionic descriptors: MW^A^, the molecular weight; nSK^A^, the number of non-H atoms; nBO^A^, the number of non-H bonds; SCBO^A^, the sum of conventional bonds (H-depleted); nDB^A^, number of double bonds; nO^A^, the number of oxygen atoms; nS^A^, the number of sulfur atoms; nF^A^, the number of fluorine atoms; and nHet^A^, the number of heteroatoms. They all describe the size of ions, excluding hydrogen atoms from the description. Therefore, it is readily apparent that ILs with a high content of non-hydrogen atoms, with particular emphasis on oxygen, sulfur and fluorine, also indicate higher values ​​of PC2. In case of PC2, cations were not recognized as having a major influence on its values.

While analyzing the scatter plot, it can be seen that within the space of constitutional indices, the ILs form distinct, separate clusters, what was also confirmed by HCA results. We have marked the clusters as different shapes (Fig. 2:II, III). We compared liquids from different clusters with each other by their structure. It was observed that the ILs possesses several common features, which give each cluster a unique character. Our observations were in good accordance with our previous interpretation of the PC’s meaning. The descriptions of the formed clusters are as follows: cluster **A:** contains all the ILs consisting of relatively small cations (such as imidazolium, pyridinium, pyrrolidinium) and halogen ions; cluster **B:** cations of similar size to cations from cluster A but with larger anions (tetrafluoroborate, hexafluorophosphate, ions containing sulfur); cluster **C:** ILs with cations similar to those from clusters A and B, all containing bis(trifluoromethylsulfonyl)imide (TFSI) as an anion; cluster **D**: ILs with large cations, mostly ammonium and phosphonium, with long alkyl chains attached and in which anions are mostly amino acids, although some are also similar to those of cluster B; cluster **E:** ILs with large phosphonium cations with long alkyl chains and large amino acid anions; and cluster **F**: ILs with large cations and halogens as anions.

### Exploration of the physicochemical properties of ILs

**Fig. 4:**
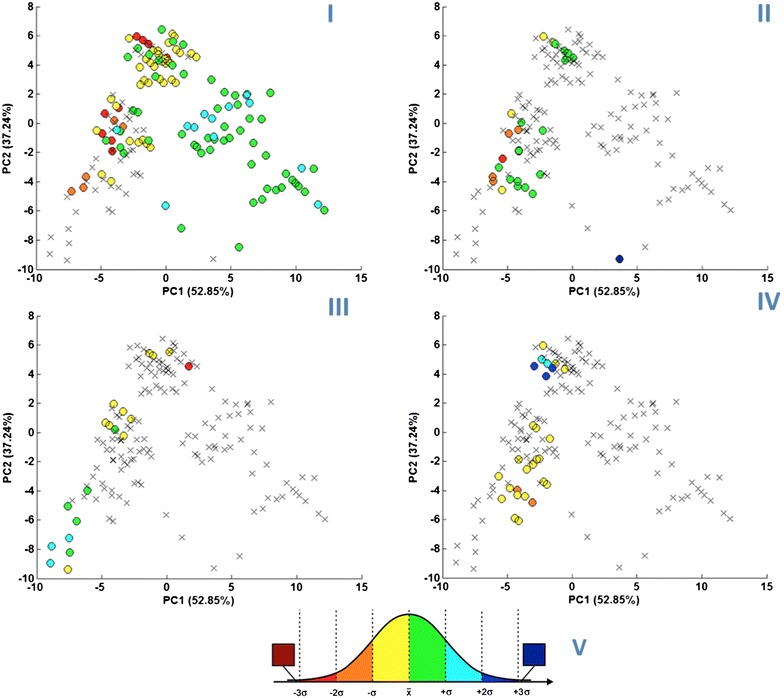
Scatter plots representing 17 ILs in the space of PC1 and PC2. *Colored dots* represent the values of the analyzed property: **I** viscosity, **II** K_OW_, **III** solubility in water, **IV** enthalpy of fusion, **V** scheme of color ranges used to represent the standardized physicochemical properties of ILs

We followed the detailed description of the PCA results with an approach in which each value of the specific physicochemical properties was a colored dot on the plot of PC1 vs. PC2 (Fig. 4:I–IV, where each color corresponds to the standardized values of the tested property, Fig. 4:V). This methodology was intended to demonstrate the change in the value of a property in the area of structural changes. For a detailed description of this method, please refer to the Methodology section. The findings yielded by this approach (represented in Fig. 4:I–IV) along with the simple theoretical explanation are described below. These general conclusions are also a key to interpret the results obtained with the ILPC. All the remarks are helpful with the understanding of the ILPC final performance.

#### Exploration of ILs’ viscosity in the space of constitutional descriptors

Viscosity (Fig. 4:I) is the physicochemical property for which we had the widest set of literature data available. Therefore, the trends in this plot are the most readily visible. Previous reports suggest that the viscosity of an IL is strongly connected to its intermolecular forces, which depend mainly on hydrogen bond formation [20]. Unfortunately, this type of intermolecular relation is not covered by our set of molecular descriptors. However, the results (trends of the viscosity values) that we observed were very satisfying. Our first observation is that the viscosity rises with increasing PC1 values and decreasing PC2 values. From this information, we infer that larger cations and anions with more saturated and longer alkyl chains create more viscous ILs. Larger alkyl chains have strong affinity for each other and are more likely to create hydrogen bonds. Although constitutional indices were used as molecular descriptors, we do not directly refer to intermolecular forces; instead, we treat their relation to cation size as a simplified but reasonable explanation. Our results are also in good accordance with previous experimental findings, suggesting that long alkyl chains increase IL viscosity due to significantly increased steric hindrance [21–23]. In addition, it has been previously reported that the TFSI anion also reduces the viscosity of ILs [23]. This finding was confirmed by our results, in which cluster C (TFSI-containing ILs) appears to exhibit the lowest viscosity.

The IL’s assignment to the particular cluster can also be a useful indicator suggesting its viscosity value. ILs that belong to the clusters A, B or C are more likely to indicate lower viscosity values than ILs from clusters D, E or F.

#### Exploration of ILs’ K_OW_ in the space of constitutional descriptors

The n-octanol–water partition coefficient (K_OW_) (Fig. 4:II) was the second physicochemical property that we tested in this work. This property, which is defined as the ratio of a compound’s solubility in polar and nonpolar solvents, is directly connected to the solvation process. In general, solvation depends mostly on intermolecular forces, such as electrostatic and van der Waals forces or hydrogen bond formation [24].

As in the previous case (viscosity), our descriptors do not directly describe the ability of particles to interact with each other; therefore, no information regarding the intermolecular forces that arise in the IL are delivered by constitutional indices. However, we can try to relate the descriptors from our set to the more complex properties of the molecules, thereby indirectly describe ionic interactions. As previously noted, PC1 is strongly connected to the number of carbon atoms in sp^3^ hybridization and the number of hydrogen atoms in both ions. This suggests that the value of PC1 is higher for molecules that contain long, saturated alkyl chains. The presence of alkyl chains as substituents in the ions suggests that they will have greater hydrophobic characteristics and therefore indicates stronger attraction to nonpolar solvents, such as n-octanol, due to electrostatic forces. Increasing the length of the alkyl chains also reduces any disproportionality of charge distribution in the molecule. As a result, ion solvation by the polar solvent’s particles will be weakened, reducing the solubility of the ILs in polar solvents.

Although the range of K_OW_ values collected from the literature was smaller than the values obtained for the viscosity, a noticeable trend can nonetheless still be seen in the changes. We observed that values of K_OW_ changes proportionally to the values of PC1. For increasing cation/anion size, the corresponding polarity decreases (the charge is less influent, i.e., more delocalized); in addition, the cation/anion possesses greater hydrophobic characteristics, so the value of K_OW_ increases. Our conclusions are in good accordance with previous findings [25–27].

#### Exploration of ILs’ solubility in the space of constitutional descriptors

Third analysis was conducted to assess the ILs’ water solubility (Fig. 4:III). As expected, the trend observed for changes in aqueous solubility is opposite to that of K_OW_. Although the availability of experimental values for this property was rather small, the trends that we observed were very explicit. As in the previous case (K_OW_), PC1 was found to differentiate the ILs by solubility. Additionally, the influence of PC2 is also noticeable. Solubility is basically governed by the same phenomenon that K_OW_ coefficient is. As mentioned before, the dissolution process is mostly connected to electrostatic forces, van der Waals forces and hydrogen bond formation. Smaller ions are usually less hydrophobic than long ones; therefore, polar solvents, such as water, can better dissolve small-molecule ILs. This trend is very well described by PC1. In addition, based on theoretical calculations, Zhou et al. [28] grouped anions constituting ILs by their contribution to IL solubility in water. In our analysis, the solubility trend described by PC2 (which defines the structures of the anions) is very similar to the findings presented by these authors. The smallest values of water solubility were found for ILs in cluster A (the lowest values of PC2, ILs containing halogen atoms) through cluster B (middle PC2 values, average sized anions containing nitrogen and sulfur) to cluster C (highest values of PC2, only TFSI anions). Also in this case, our simplified approach to structural description yielded satisfying results for the physicochemical property analysis.

#### Exploration of ILs’ enthalpy of fusion in the space of constitutional descriptors

The enthalpy of fusion (Fig. 4:IV) is a property that describes how much energy is needed to transform a solid-state compound into a liquid state. Similar to every other property taken into account in this study, it is directly related to the intermolecular relations between ions constituting ILs. The value of **Δ**H_F_ is normally considered to be the effect of molecular packing in crystals [29] or hydrogen bond formation [30]. Due to the reasons discussed earlier (the specific nature of the descriptors used here), we can only provide a simplified explanation of the trends observed in our data set. We found that ionic liquids consisting of larger, more complex ions were recognized to have higher values of **Δ**H_F_. This time, the trends were mostly dependent on the values of PC2, which describes anion size. On the linear map, ILs with the highest values of **Δ**H_F_ are located in cluster C, while those with the smallest values of **Δ**H_F_ are located in cluster A.

The size of the anion in a particular IL is usually smaller than that of the cation. Because changes in anion size appear to follow the trend of **Δ**H_F_ change, this may suggest that with decreasing dissimilarity among ion sizes, the distribution of each ion within the IL becomes more organized, while the energy required for transformation into an irregular manner of liquid phase increases. This could also be connected to the IL’s decreasing ability to create hydrogen bonds under such conditions, as observed in Zhou et al. [28].

#### Further exploration of the trends with use of the theoretically derived data

During the initial stage of our work, we dealt with modest data accessibility for three out of four properties of our interest (K_OW_, S and **Δ**H_F_). In order to verify, if our findings based on the limited amount of experimental data are correct, we additionally performed a further analysis basing on the computationally derived information about IL’s properties.

We employed three QSPR models in order to fulfill the lacking information. For the K_OW_ prediction, we employed the model previously developed and published by our team [31]. Solubility predictions (here expressed as a mole fraction) were based on the model developed by Freire et al. [25]. The third model—allowing for **Δ**H_F_ predictions for ILs, was developed as a part of this work due to the lack of similar models in the literature (see Supporting Material for details concerning QSPR models). In case of K_OW_ and **Δ**H_F_, we verified the reliability of predictions by analyzing the tested ILs relation with model’s applicability domain—AD (which is a theoretical space containing compounds for which the predictions are most plausible). We were however unable to determine solubility model’s AD. To overcome this problem we decided to verify the model’s performance differently. We compared the results from solubility predictions with all the predictions obtained for K_OW_, assuming that the modeled values should indicate opposite trends. Knowing that both QSPR models were developed for different set of ILs and with use of different set of molecular descriptors, opposite trends would confirm well performance of both models for the entire dataset. The results were indeed satisfactory; therefore we used all data predicted by K_OW_ model and S model to the further analysis.

**Fig. 5:**
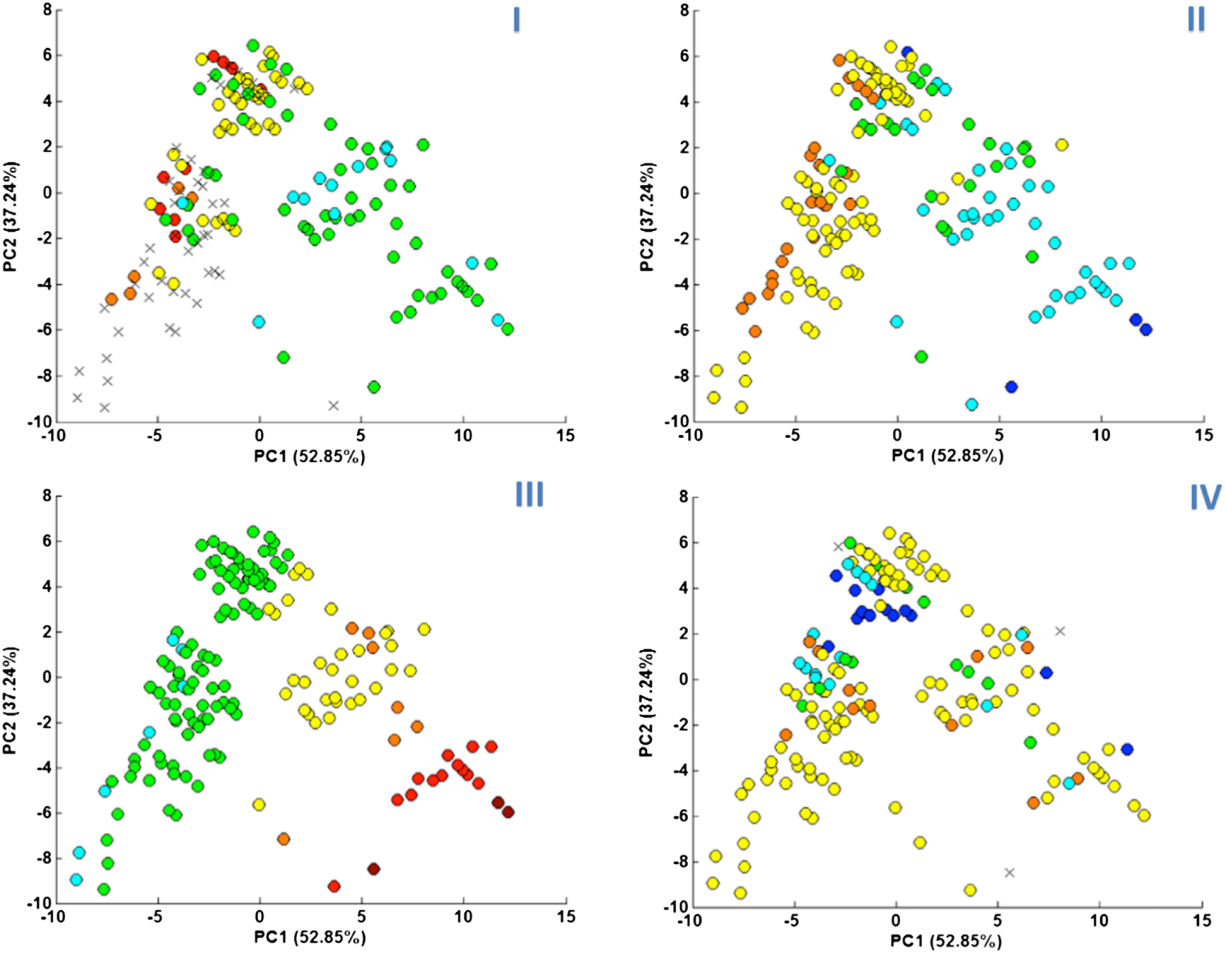
Scatter plots representing 172 ILs in the space of PC1 and PC2 based on the enhanced set of data. *Colored dots* represent the values of the analyzed property: **I** viscosity, **II** K_OW_, **III** solubility in water, **IV** enthalpy of fusion

Figure 5:I–IV shows the results of trends analysis performed on the enhanced data set. In both cases of K_OW_ and S, the trends identified on the basis of the experimental results are in the agreement with trends identified on the computationally derived data (Fig. 5:II, III). Both the K_OW_ and the S value’s changes are proportional to the values of the PC1. Moreover, they exhibit opposite correlation with PC1, as expected. PCA performed on the experimental data of solubility also indicated a visible trend of change with respect to PC2. In the case of analysis performed on computational data, this trend cannot be easily noticed. This is because the area of the plot covered by ILs with experimental data available (clusters A, B and C) is now covered by ILs with data classified mostly to the same range and having the same color on the plot. For the ILs with PC1 values higher than about 0, the mentioned trend seems to be opposite. This is the area covered by ILs containing big cations. We think that the influence of cation is dominating for those ILs and therefore ILs from clusters D, E and F are not in the agreement with the PC2 dependent trend noticed for experimental data. The trend of K_OW_ change is more noticeably dependent on PC2 for the experimental data (opposite to solubility trend as expected) but not really noticeable for computational data. In the second case, each values range (marked as a separate color) seems to cover an entire range of PC2 values. This may indicate that cation’s influence on K_OW_ is bigger than on solubility, and therefore property’s dependence on PC2 (that describes mostly anion’s size) is not visible for K_OW_. For the last property (**Δ**H_F_—Fig. 5:IV), the trend was slightly different than in our previous findings however still revealed dependence of the of the **Δ**H_F_ values on the IL’s assignment to a specific data cluster. Basing on those results we can conclude that among ILs, those from cluster C are more likely to have a higher **Δ**H_F_ value. It also seems that ILs with halogen as an anion never reaches relatively high **Δ**H_F_ values. Therefore this is less likely for ILs form cluster A or F to indicate high enthalpy of fusion. ILs from cluster B, D or E has both low and high **Δ**H_F_ values of and it is difficult to find a trend for them. Nevertheless, although we did not confirm previous findings we still consider the information we extracted as valuable only with the smaller certainty.

#### Exploration of the physicochemical properties of ILs: a summary

**Fig. 6:**
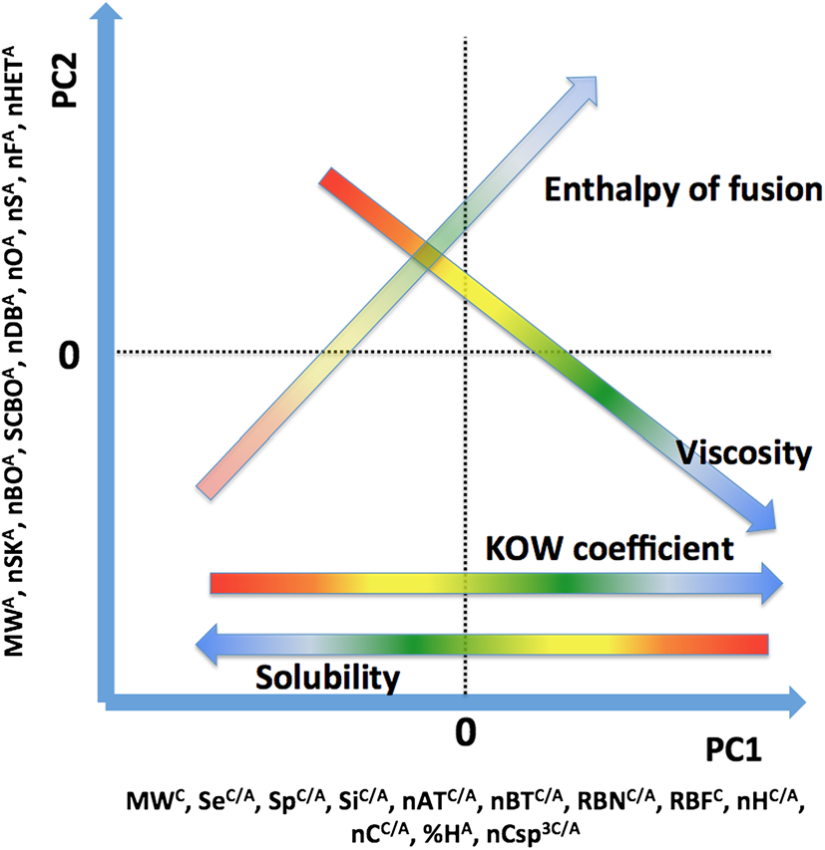
Diagram representing changes observed in the physicochemical properties of the ILs, within the space of two PCs

Most of the results reported here are in good agreement with our expectations based on theoretical knowledge [21–23, 25, 26, 28], but the main achievement is that these observations are now related to a simple measure, the size of the ions of an IL. The various aspects of an IL structure that are known to influence its properties are now simplified to one common measure. As a result, after exploring the trends for each of the four physicochemical properties, it was possible to present the findings in the corporate plot shown in Fig. 6. The fact that all these physicochemical properties yielded satisfying results (noticeable trends) for the same set of descriptors has created additional opportunities: as seen in Fig. 6, it is possible to perform only one analysis to summarize four different physicochemical properties of the studied ILs.

### Ionic Liquid PhysicoChemical (ILPC) predictor: a tool for the initial screening of IL properties

All chemometric methods are tools for providing additional information that otherwise is concealed under unnecessary and unwanted “informational noise”. Uncovering initially unnoticeable facts, as well as simplifying the dimensionality of the problem, is what chemometrics are primarily used for, we were finally managed to develop the ILPC tool. As mentioned before, it is designed to provide additional specific information about ILs based on the simplest information we can extract from the structure of the IL.

**Fig. 7:**
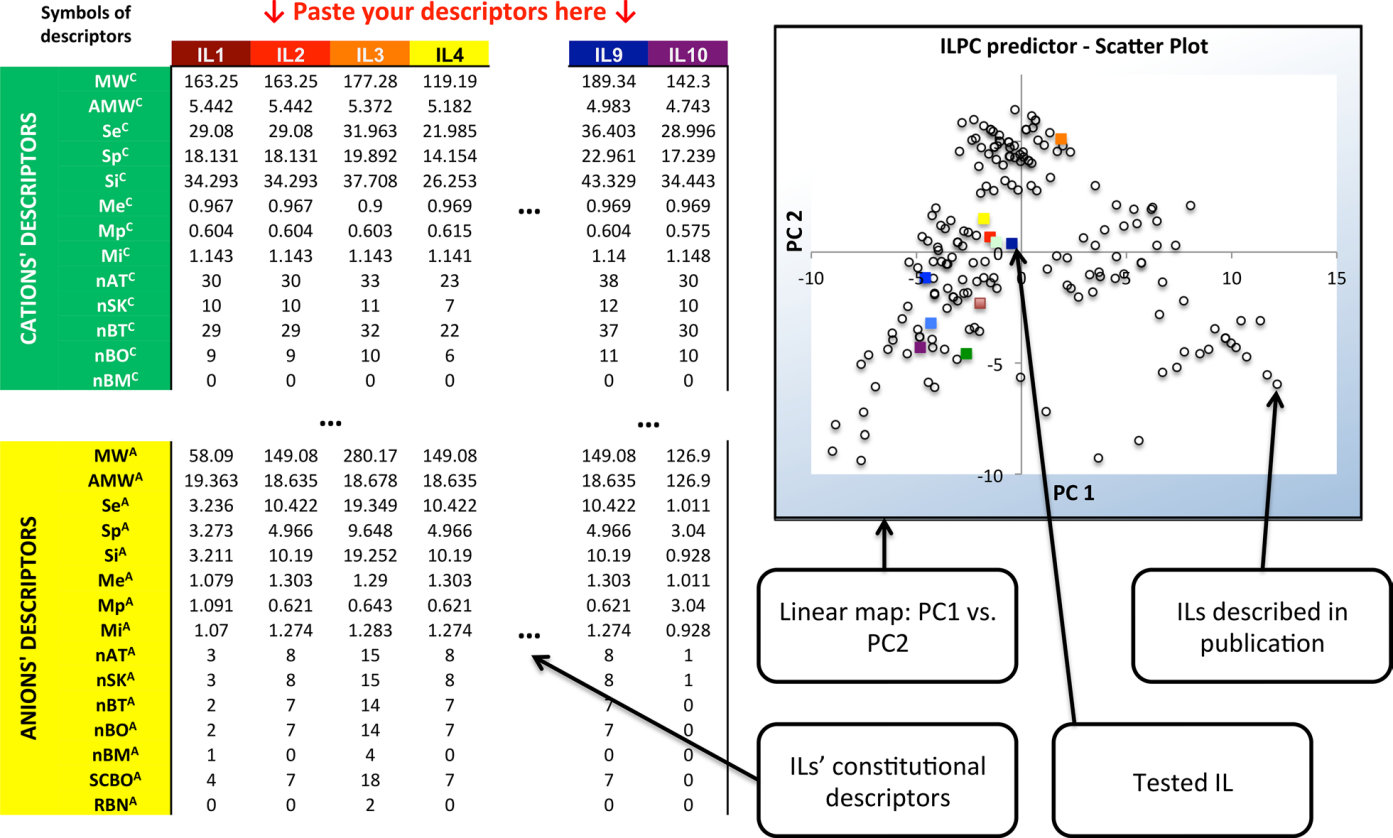
An example of ILPC performance

The ILPC predictor we present is a simple tool for predefining the physicochemical properties of an IL. Based on the molecular formula of the ions constituting ILs, one can calculate the set of constitutional descriptors. This is the first advantage of using ILPC: because 0D constitutional descriptors are easy to obtain, there is no need for the user to have precise knowledge of the calculation used for molecular indices. Such descriptors can thereafter be used to present the tested IL in the space of the 1st versus the 2nd PC on an ILPC scatter plot, which is the main deliverable of the proposed tool. Figure 7 provides an example of the ILPC’s performance. Using this paper as an interpretation guideline, one can define the properties of the tested IL and then classify it into one of the 6 structural clusters, qualitatively answering questions regarding its expected properties, such as the viscosity, n-octanol–water partition coefficient, solubility and enthalpy of fusion. Moreover, one can use this tool to compare ILs with each other. Basically, 10 slots are available for the comparison of different ILs, although the tool’s options can be extended to cover any number that the user requires. The methodological procedure that should be followed when using this tool is very simple (see the Additional file 1 for details).

ILPC can be very useful in the IL design process. With no need of experimental characterisation of particular IL to predict its properties by ILPC, one can select several sets of different ionic combinations and then choose the IL with the properties closest to those desired. In addition, Fig. 6 contains names of descriptors having the major influence on PCs’ values. One can use this information as a guideline, indicating which particular feature of IL should be kept on high/low level in order to create an IL characterized by desired properties. This particular use of ILPC is the most important because it allows for greater experimentalist awareness in the synthesis of ILs.

The complete guidelines for the use of ILCP are given in the Additional file 1. The SM also contains all the measures needed for the ILCP tool to perform properly.

## Conclusions

In this work, we have presented a tool for qualitative assessment of the physicochemical properties of ILs that relies only on the chemical formulas of the ions. The applied approach showed that there are clear trends for the varying values of different physicochemical properties (i.e., the viscosity, n-octanol–water partition coefficient, solubility and enthalpy of fusion) depending on the size of the ions, their degree of saturation, their nature (organic/inorganic) and the contents of the various elements in the anions (other than H, mostly sulfur and fluorine). We created a linear map that presents the mutual relationship within a wide range of ILs while maintaining a very high degree of variation (90.09 %). Additionally, our findings concerning the structure-properties relationship based on the experimental data were also confirmed by analysis performed with use of the computationally derived data.

Although the obtained results mainly confirmed our theoretical presumptions, they also made possible the deduction that in the case of these four characteristics, the impact of the ions is additive rather than opposite (PC1 and PC2 were positively correlated with the descriptors). In addition, the graphical presentation of such results can better expose the relationship between different groups of ILs, while systematizing knowledge about their properties.

The most important feature of the tool is that it is possible to carry out the analysis without carrying out experiments or even synthesizing the target IL. The only required information is the chemical formula of the two ions constituting the IL. Moreover, the ILPC predictor allows for simultaneous comparisons of a greater number of ILs with each other, so if one tries to obtain a liquid with the assumed characteristics, then the entire set can be analyzed, allowing the investigator to choose the IL that will best suit the particular needs of the application. The main obstacle standing in the way of ILPC improvement is the lack of satisfying amounts of experimental data describing IL properties. In the future, modification of the current version of ILPC is planned as the availability of new data increases. New data will be included in the analysis; thus, the obtained trends will be described as a function of the particular characteristics of the structure and will become increasingly more accurate and reliable. With the development of these tools, we hope to eventually move from qualitative descriptions of selected properties to their quantitative description.

## Methods

### Experimental data

The data used in our work were drawn from the database developed in an earlier stage of our project (http://db.qsar.eu.org) [32]. This database was created based on experimental data available in the literature and other open-access databases. It contains information about different IL properties, both physicochemical and toxicological. When filling the database, only high-quality experimental data were utilized (results for a series of ILs that have been obtained under the same conditions and with the use of the same protocol). In addition, all entries in our database were evaluated according to the Klimish scale to determine their reliability [33]. To each entry, we assigned a different range (from 1, highest reliability, to 4, lowest reliability). The data used in this work had a quality no lower than 3.

**Fig. 8:**
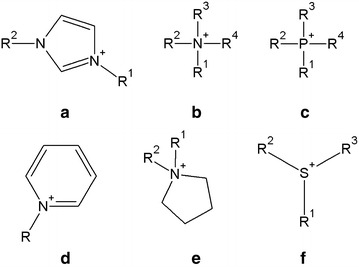
Schematic structures representing cations of each IL sub-group tested in this work: **a** imidazolium, **b** ammonium, **c** phosphonium, **d** pyridinium, **e** pyrrolidinium, and **f** sulfonium

In this study, we used a set of 172 ILs, consisting of six different types of cations (namely, imidazolium, ammonium, phosphonium, pyridinium, pyrrolidinium and sulfonium; see Fig. 8) and 38 different anions (halogens, fluor-based, sulfur-based, amino acid-like, etc.; list of ions constituting all tested ILs can be found in the Additional file 1). We focused on four different physicochemical properties: the viscosity [η], n-octanol–water partition coefficient [log K_OW_], solubility [S] and enthalpy of fusion [**Δ**H_F_]. The number of data points for each IL sub-group is presented in Table 1. A complete list of all 172 ILs investigated in this study can be found in the Additional file 1.

### Optimization of IL’s structures

Our approach assumed the sufficient performance of PCA, which is described in detail in the next paragraph. It is important to note that similar to any other mathematical technique, PCA can only be performed on numerical data. To analyze structural similarities/differences between each IL and compare these findings with different properties of interest, we first had to translate their structures into numerical values. This type of translation is widely used in many theoretical studies [34–38]. It involves the calculation of so-called “molecular descriptors”.

Each molecular descriptor is a solution of a mathematical formula, in which information about the molecule’s properties (such as molecular weight, coordinates of atoms in space, structural conformation, etc.) are used as variables. Each descriptor is unique and provides different information about the molecule.

First, we created a virtual representation of both the cations and anions in every IL (of 172 total) using the MOLDEN [39] software. The files containing the 3D representations of molecules were thereafter subjected to an optimization process. The optimization process is a crucial step for calculating classes of 3D and 4D descriptors because they are based on the spatial distribution of atoms in the molecule [40]. This approach also grants repeatability to the calculations, reducing the random differences between virtual structures developed by different experimentalists or by using different programs. In this step, quantum–mechanical calculations were performed to find the spatial conformation with the lowest internal energy of the molecule. The structures of the cations and anions were optimized separately for each IL. We ran all our optimization calculations in MOPAC2012 [41] software, using the PM7 semi-empirical method [42]. Thereafter, we used DRAGON [43] software to calculate different sets of molecular descriptors. Basing on the optimized structures from the previous step, we obtained 2924 different descriptors for each IL.

### Principal component analysis

Principal Component Analysis is a very popular mathematical technique, most commonly used to reduce the dimensionality of an analyzed dataset. Here, we present the general idea of PCA. Further information about this technique can be found elsewhere [44–47].

Principal Component Analysis is based on the observation that some of the features (in our case, molecular descriptors) that describe samples/cases (in our case, ILs) are correlated with each other, and according to that fact, those features carry the same information about the samples. In the initial data matrix, we can treat each feature as a separate vector, adding the same amount of information to the overall description of the samples. This amount of information is expressed as variance, and it is equal to 1 for each initial feature. By performing PCA, we can mathematically develop new, artificial features called Principal Components (PCs), which are eigenvectors of the covariance matrix, calculated from the original data matrix. In this manner, we can treat PCs as compositions containing some fragments of all initial features or, in the other words, a mixture of initial features in different proportions. The biggest advantage of PCA is that the first PC always contains the greatest amount of information (variance) that can possibly be described by one feature in the analyzed data matrix. Each subsequent PC contains the greatest amount of information not explained by the previous PC. Thus, (I) the variance of PCs can extend to a value greater than 1 (more information than any initial feature), and (II) PCs are arranged by descending variance values.

In this work, we used the PCA approach to group the studied ILs based on their structural similarity and to then identify which aspect of the structure is actually responsible for a given IL physicochemical property, as well as how its modification impacts this property. To achieve an effective presentation of IL structure in the descriptor space, we chose only the first and second PCs (score plot), in accordance with the demonstrative criterion [48]. Taking only the first two PCs for further analysis, we reduced the number of features, taking care to preserve as much information as possible. The physical interpretation of PC1 and PC2 was defined using Malinowski’s rule [49]. Within the space of the first and second PCs, we were able to create clusters of objects (ILs) (we assume that the ILs located close to each other in the projection were structurally similar). For our calculations, we used MATLAB (R2013b 8.2.0.701) software [50].

### Hierarchical cluster analysis

HCA is a grouping method, which allows arranging the tested objects into clusters, basing on the mathematically derived distances between them. Selecting appropriate distance measure and clustering technique define the exact purpose of analysis. In our work we performed HCA on ILs represented on the linear maps, in order to provide some detailed information concerning their distribution. We used Euclidean distance and Ward’s clustering method. For calculations, we used MATLAB (R2013b 8.2.0.701) software [50].

### Physicochemical properties representation

To perform the analysis, we transferred the collected physicochemical data into a range scale, in which the ranges corresponded to the standardized values of the tested property. Then, we assigned colors to the ranges (Fig. 4:V) and, after that, colored markers representing particular ILs on the PCA-derived score plots. This arbitrary operation was applied in order to help identifying the trends in the data distribution. Structurally driven grouping of ILs having the same distribution as the tested property (the same color markers) proves the dependence between the structure and physicochemical property.

### QSPR modeling

Collected and evaluated experimental data for enthalpy of fusion (**Δ**H_F_) of 30 ILs were split into the training (to develop the QSPR model) and validation (to examine the model’s ability to predict **Δ**H_F_ for compounds other than those used for the calibration) sets [51]. A table summarizing the splitting procedure can be found in the electronic Additional file 1. The search for the optimal descriptor’s combination for QSPR modeling was carried out by applying the genetic algorithm, implemented in the QSARINS software [52, 53]. The multiple linear regression (MLR) was employed as the method of modeling. The model was developed according to the golden standards and recommendations of the Organization for Economic Co-operation and Development (OECD) [54–57]. According to those principles we have calculated the measures of goodness-of-fit, robustness and predictive ability of the developed model as well as defined the applicability domain (AD). The detailed information of the model’s parameters are presented in the Additional file 1.

## Additional file

10.1186/s13321-016-0152-4A tool developed within this work, allowing for predefining the physicochemical properties of ionic liquids. Data concerning IL’s properties and names of the descriptors used is also included in this file.

## References

[1] Zhu S, Chen R, Wu Y (2009). A mini-review on greenness of ionic liquids. Chem Biochem Eng Q.

[2] Welton T (1999). Room-temperature ionic liquids: solvents for synthesis and catalysis. Chem Rev.

[3] Sheldon RA, Lau RM, Sorgedrager MJ (2002). Biocatalysis in ionic liquids. Green Chem.

[4] Endres F (2002). Ionic liquids: solvents for the electrodeposition of metals and semiconductors. Chem Phys Chem.

[5] Berthod A, Ruiz-Ángel MJ, Carda-Broch S (2008). Ionic liquids in separation techniques. J Chromatogr A.

[6] MacFarlane DR, Tachikawa N, Forsyth M (2014). Energy applications of ionic liquids. Energy Environ Sci.

[7] Paul TC, Morshed AKMM, Khan JA (2013). Nanoparticle Enhanced Ionic Liquids (NEILS) as working fluid for the next generation solar collector. Procedia Eng.

[8] Teramoto D, Yokoyama R, Kagawa H et al (2012) A novel ionic liquid-polymer electrolyte for the advanced lithium ion polymer battery. In: Molten salts ion. Liq. Never Twain? pp 367–388

[9] Freiré MG, Santos LMNBF, Marrucho IM, Coutinho JAP (2012) Predicting the thermodynamic behaviour of water + ionic liquids systems using COSMO-RS. In: Molten salts ion. Liq. Never Twain? pp 101–121

[10] Pernak J, Sobaszkiewicz K, Mirska I (2002). Anti-microbial activities of ionic liquids. Green Chem.

[11] Latała A, Stepnowski P, Nȩdzi M, Mrozik W (2005). Marine toxicity assessment of imidazolium ionic liquids: acute effects on the Baltic algae Oocystis submarina and Cyclotella meneghiniana. Aquat Toxicol.

[12] Latała A, Nędzi M, Stepnowski P (2010). Toxicity of imidazolium ionic liquids towards algae. Influence of salinity variations. Green Chem.

[13] Frade RFM, Matias A, Branco LC (2007). Effect of ionic liquids on human colon carcinoma HT-29 and CaCo-2 cell lines. Green Chem.

[14] Jastorff B, Mölter K, Behrend P (2005). Progress in evaluation of risk potential of ionic liquids—basis for an eco-design of sustainable products. Green Chem.

[15] Bonhôte P, Dias A-P, Armand M (1996). Hydrophobic, highly conductive ambient-temperature molten salts. Inorg Chem.

[16] Ngo HL, LeCompte K, Hargens L, McEwen AB (2000). Thermal properties of imidazolium ionic liquids. Thermochim Acta.

[17] Coleman D, Gathergood N (2010). Biodegradation studies of ionic liquids. Chem Soc Rev.

[18] Das RN, Roy K (2013). Advances in QSPR/QSTR models of ionic liquids for the design of greener solvents of the future. Mol Divers.

[19] Sosnowska A, Barycki M, Zaborowska M (2014). Towards designing environmentally safe ionic liquids: the influence of the cation structure. Green Chem.

[20] García G, Atilhan M, Aparicio S (2014). Viscous origin of ionic liquids at the molecular level: a quantum chemical insight. Chem Phys Lett.

[21] Tokuda H, Hayamizu K, Ishii K (2005). Physicochemical properties and structures of room temperature ionic liquids. 2. variation of alkyl chain length in imidazolium cation. J Phys Chem B.

[22] Jacquemin J, Husson P, Padua H, Majer V (2006). Density and viscosity of several pure and water-saturated ionic liquids. Green Chem.

[23] Rajappan R, Shingade PD, Natarajan R, Jayaraman VK (2009). Quantitative structure-property relationship (QSPR) prediction of liquid viscosities of pure organic compounds employing random forest regression. Ind Eng Chem Res.

[24] Spickermann C, Thar J, Lehmann SBC (2008). Why are ionic liquid ions mainly associated in water? A Car-Parrinello study of 1-ethyl-3-methyl-imidazolium chloride water mixture. J Chem Phys.

[25] Freire MG, Neves CMSS, Ventura SPM (2010). Solubility of non-aromatic ionic liquids in water and correlation using a QSPR approach. Fluid Phase Equilib.

[26] Katritzky A, Kuanar M, Stoyanova-Slavova I (2008). Quantitative structure–property relationship studies on Ostwald solubility and partition coefficients of organic solutes in ionic liquids. J Chem Eng Data.

[27] Rybinska A, Sosnowska A, Zaborowska M (2016). Filling environmental data gaps with QSPR for ionic liquids: modeling n-octanol/water coefficient. J Hazard Mater.

[28] Zhou T, Chen L, Ye Y (2012). An overview of mutual solubility of ionic liquids and water predicted by COSMO-RS. Ind Eng Chem Res.

[29] Gharagheizi F, Gohar MRS, Vayeghan MG (2012). A quantitative structure-property relationship for determination of enthalpy of fusion of pure compounds. J Therm Anal Calorim.

[30] Peppel T, Roth C, Fumino K (2011). The influence of hydrogen-bond defects on the properties of ionic liquids. Angew Chemie Int Ed.

[31] Rybinska A, Sosnowska A, Grzonkowska M (2016). Filling environmental data gaps with QSPR for ionic liquids: modeling n-octanol/water coefficient. J Hazard Mater.

[32] ILsTOX database of ionic liquids properties (2015). University of Gdansk, Gdansk. http://db.qsar.eu.org. Accessed 15 May 2016

[33] Klimisch HJ, Andreae M, Tillmann U (1997). A systematic approach for evaluating the quality of experimental toxicological and ecotoxicological data. Regul Toxicol Pharmacol.

[34] Roy K, Das RN, Popelier PLA (2014). Quantitative structure-activity relationship for toxicity of ionic liquids to Daphnia magna: aromaticity vs. lipophilicity. Chemosphere.

[35] Mousavisafavi SM, Mirkhani SA, Gharagheizi F, Akbari J (2012). A predictive quantitative structure–property relationship for glass transition temperature of 1,3-dialkyl imidazolium ionic liquids. J Therm Anal Calorim.

[36] Mirkhani SA, Gharagheizi F, Farahani N, Tumba K (2013). Prediction of surface tension of ionic liquids by molecular approach. J Mol Liq.

[37] Mirkhani SA, Gharagheizi F, Ilani-Kashkouli P, Farahani N (2012). Determination of the glass transition temperature of ionic liquids: a molecular approach. Thermochim Acta.

[38] Ma S, Lv M, Deng F (2015). Predicting the ecotoxicity of ionic liquids towards Vibrio fischeri using genetic function approximation and least squares support vector machine. J Hazard Mater.

[39] Schaftenaar G, Noordik JH (2000). Molden: a pre- and post-processing program for molecular and electronic structures. J Comput Aided Mol Des.

[40] Toropov A (1999). Ideal symmetry method for 4D models: application for QSPR studies of alkanes and alkylbenzenes. J Struct Chem.

[41] Stewart JP (2012) MOPAC, stewart computational chemistry. Colorado Springs, CO, USA J. MOPAC

[42] Stewart JJP (2013). Optimization of parameters for semiempirical methods VI: more modifications to the NDDO approximations and re-optimization of parameters. J Mol Model.

[43] DRAGON (software for molecular descriptor calculation) (2014), version 6.0-2014, Talete srl., Milano. http://www.talete.mi.it

[44] Jolliffe IT (2002). Principle component analysis.

[45] Zuba D, Parczewski A (2008). Chemometria w Analityce.

[46] Praus P (2005). SVD-based principal component analysis of geochemical data. Cent Eur J Chem.

[47] Abdi H, Williams LJ (2010). Principal component analysis. Wiley Interdiscip Rev Comput Stat.

[48] Mazerski J (2004) Podstawy chemometrii, 1st ed. Wydawnictwo Politechniki Gdańskiej, 2000, Gdańsk

[49] Pack S (1991) Factor analysis in chemistry (2nd edn), E. R. Malinowski, Wiley-Interscience. J Chemom 5:545. doi:10.1002/cem.1180050607

[50] MATLAB with Statistics Toolbox Release 2012b (2013). Mathworks Inc., Natick, Massachusetts, United States. http://www.mathworks.com/products/matlab/

[51] Gramatica P, Pilutti P, Papa E (2004). Validated QSAR prediction of OH tropospheric degradation of VOCs: splitting into training-test sets and consensus modeling. J Chem Inf Comput Sci.

[52] Gramatica P, Chirico N, Papa E (2013). QSARINS: a new software for the development, analysis, and validation of QSAR MLR models. J Comput Chem.

[53] Gramatica P, Cassani S, Chirico N (2014). QSARINS-chem: insubria datasets and new QSAR/QSPR models for environmental pollutants in QSARINS. J Comput Chem.

[54] OECD The report from the expert group on (quantitative) structure activity relationship [(Q)SARs] on the principles for the validation of (Q)SARs. Series on testing and assessment No. 49 (ENV/JM/MONO(2004)24). Organisation of Economic Cooperation and Development, Paris, France (2004)

[55] OECD Guidance Document on the Validation of (Quantitative) Structure Activity Relationships [QSAR] Models. Organisation for Economic Cooperation and Development, Paris, France (2007). doi:10.1787/9789264085442-en

[56] Gramatica P (2007). Principles of QSAR models validation: internal and external. QSAR Comb Sci.

[57] Tropsha A, Gramatica P, Gombar VK (2003). The importance ofbeing earnest: validation is the absolute essential for successfulapplication and interpretation of QSPR models. QSAR Comb Sci.

[58] Todeschini R, Consonni V (2000) Handbook of molecular descriptors. WILEY-VCH Verlag GmbH, D-69469 Weinheim SBN 3-52-29913-0

